# mScarlet and split fluorophore mScarlet resources for plasmid-based CRISPR/Cas9 knock-in in *C. elegans*

**DOI:** 10.17912/micropub.biology.000871

**Published:** 2023-06-14

**Authors:** Gillian Witten, Ella DeMott, George Huang, Francis Zelasko, Bailey de Jesus, Chandi Mulchand, Liam Schuck, Stephen Pullman, Amelie Perez, Priya Mahableshwarkar, Zheng Wu, Eric Andrew Cardona, Jonathan T Pierce, Daniel J Dickinson, Ryan Doonan

**Affiliations:** 1 Glow Worms Stream, Freshman Research Initiative, College of Natural Sciences, The University of Texas at Austin, Austin, Texas, USA; 2 Department of Neuroscience, The University of Texas at Austin, Austin, Texas, USA; 3 Department of Molecular Biosciences, The University of Texas at Austin, Austin, Texas, USA

## Abstract

Fluorescent proteins allow the expression of a gene and the behavior of its protein product to be observed in living animals. The ability to create endogenous fluorescent protein tags via CRISPR genome engineering has revolutionized the authenticity of this expression, and mScarlet is currently our first-choice red fluorescent protein (RFP) for visualizing gene expression *in vivo* . Here, we have cloned versions of mScarlet and split fluorophore mScarlet previously optimized for *C. elegans* into the SEC-based system of plasmids for CRISPR/Cas9 knock-in. Ideally, an endogenous tag will be easily visible while not interfering with the normal expression and function of the targeted protein. For low molecular weight proteins that are a fraction of the size of a fluorescent protein tag (e.g. GFP or mCherry) and/or proteins known to be non-functional when tagged in this way, split fluorophore tagging could be an alternative. Here, we used CRISPR/Cas9 knock-in to tag three such proteins with split-fluorophore wrmScarlet: HIS-72, EGL-1, and PTL-1. Although we find that split fluorophore tagging does not disrupt the function of any of these proteins, we were unfortunately unable to observe the expression of most of these tags with epifluorescence, suggesting that split fluorophore tags are often very limited as endogenous reporters. Nevertheless, our plasmid toolkit provides a new resource that enables straightforward knock-in of either mScarlet or split mScarlet in *C. elegans.*

**Figure 1. Analysis of HIS-72 , EGL-1 , and PTL-1 split wrmScarlet tags in C. elegans:**
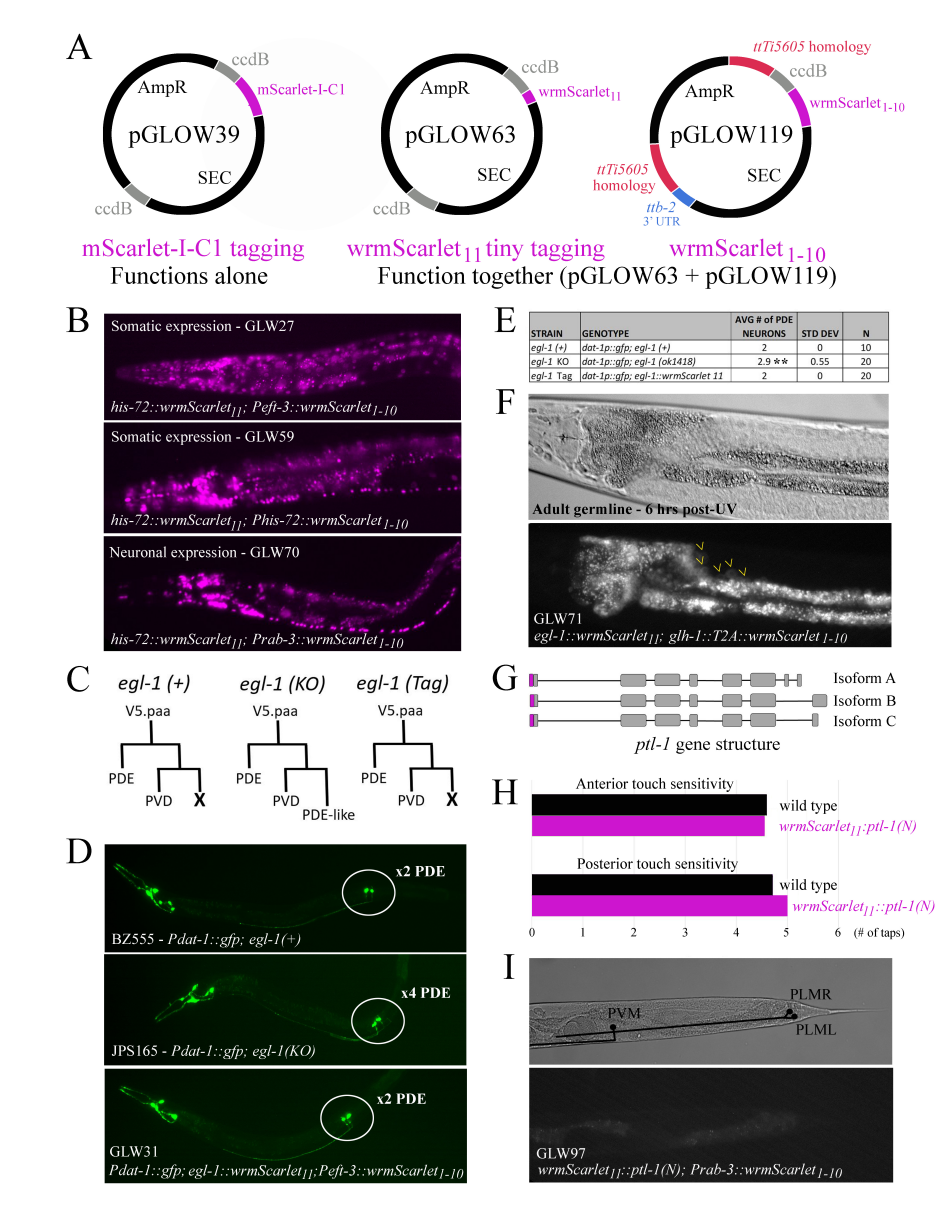
*(A)* Plasmid maps for the mScarlet knock-in constructs developed as part of this project. pGLOW39 and pGLOW63 allow tagging of any gene of interest (GOI) with mScarlet-I-C1 or split fluorophore wrmScarlet _11_ , respectively. ccdB regions are removed by DNA digest and homology arms for the GOI are cloned as described by Dickinson *et al* . 2015. pGLOW119 allows cloning of any promoter upstream of split fluorophore *wrmScarlet _1-10_* , and this construct can be inserted via CRISPR/Cas9 homologous recombination into the *ttTi5605* locus on LGII. *(B)* Testing the split fluorophore system using HIS-72. (Top) Endogenous expression of tiny-tagged HIS-72 ( *his-72::3xMyc::wrmScarlet _11_* in an *muIs252 (Peft-3::wrmScarlet _1-10_ )* genetic background. HIS-72 is visible in all somatic nuclei, consistent with Goudeau *et al* . 2021. This confirms that the 3xMyc epitope tag does not interfere with complementation of the split fluorophore. (Middle) We created an alternative somatic expression construct for *wrmScarlet _1-10_* using the *his-72* promoter and *tbb-2* (3’UTR) rather than the *eft-3* promoter and *unc-54* (3’UTR). Both somatic *wrmScarlet _1-10_* constructs appear to have ubiquitous expression in all nuclei (compare top and middle panels). (Bottom) We also created a construct for neuronal expression of *wrmScarlet _1-10_* using the *rab-3* promoter. Note that HIS-72::wrmScarlet _11_ and wrmScarlet _1-10_ complementation is visible in the neuronal nuclei of the head and ventral nerve cord. *(C) egl-1* functions within the V5.paa lineages to eliminate the sister cell of PVD via programmed cell death. *(D)* The EGL-1 tiny tag is functional. We confirmed that the PVD sister cells undergo apoptosis in animals expressing *egl-1::wrmScarlet _11_* and *Peft-3::wrmScarlet _1-10_* . The dopaminergic neuron marker *Pdat-1::gfp* was used to identify PDE neurons and “undead” PVD sister cells in L3 worms. The relevant cell bodies are circled in white. *(E)* Animals with wild type EGL-1 or tiny-tagged EGL-1 have just 2 PDE neurons, whereas *egl-1* knockout worms have an average of 3 (but sometimes 4) PDE and PDE-like neurons (p<0.0001, unpaired t-test). *(F)* To observe EGL-1 tiny tag expression, we induced programmed cell death in germ cells using UV irradiation. At 6 hours post-UV, we were unable to observe any expression in the germline (mitotic region nor death zone). Yellow arrowheads indicate out-of-focus, round, autofluorescent gut granules (i.e. halo effect). *(G)* N-terminal tiny-tagging strategy for the *ptl-1* gene. Isoforms A-C share a common first exon, so we opted to do a 5’ knock-in of *wrmScarlet _11_* at this exon to simultaneously tag multiple isoforms of PTL-1. *(H)* Mechanosensation indicates the PTL-1 tiny tag is functional. Animals bearing tiny-tagged PTL-1(N) responded to gentle touch just as well as animals bearing wild-type PTL-1 (p>0.05, paired t-tests). *(I)* PTL-1 is expressed in the axons of posterior mechanosensory neurons PVM, PLMR, and PLML. (Top) DIC image of the tail of a young adult worm, with the structures of PVM, PLMR, and PLML superimposed diagrammatically in black. Circles represent the cell bodies, whereas lines represent the axons. (Bottom) We were unable to observe any PTL-1(N) expression in the posterior mechanosensory neurons of *wrmScarlet _11_ ::ptl-1(N); Prab-3::wrmScarlet _1-10_* animals using epifluorescence microscopy at 40x, the highest resolution available in our laboratory.

## Description

mScarlet is currently our first-choice red fluorescent protein for visualizing gene expression *in vivo* (Bindels *et al.* 2017). Compared to other RFPs like mCherry or mKate2, mScarlet is less prone to unwanted oligomerization and yields superior brightness and photostability (Bindels *et al.* 2017). mScarlet was subsequently optimized for *C. elegans* expression by two independent research groups as wrmScarlet (El Mouridi *et al.* 2017) or mScarlet-I-C1 (Dickinson *et al.* 2017; Dickinson *et al.* 2018). Although wrmScarlet and mScarlet-I-C1 are both available via Addgene, the only available version of the former lacks synthetic introns to improve expression and the latter is available only as part of the SapTrap system for CRISPR/Cas9 knock-in (pZCS16 and pMS050, respectively; Schwartz and Jorgensen 2016; Dickinson *et al.* 2018; Stevenson *et al.* 2020). Thus, to expand the mScarlet toolkit in *C. elegans* , we cloned mScarlet-I-C1 into the self-excising cassette (SEC) ccdB-based vector system for CRISPR/Cas9 knock-in (Dickinson *et al.* 2015), and this plasmid is now available via Addgene as pGLOW39 ( Fig. 1A ). We have tagged several proteins with mScarlet-I-C1 with excellent results and some of these strains are already available via the CGC.

Despite the improved performance of contemporary monomeric fluorophores like mScarlet and mNeonGreen (Heppert *et al.* 2016; Bindels *et al.* 2017), sometimes the relatively large size of these tags can disrupt the function of the targeted protein. In this situation, a split fluorophore approach can be utilized, where the functional fluorescent protein tag is asymmetrically split into a small polypeptide that serves as the tag (i.e. “tiny tag”) and a large protein fragment that is conditionally disabled by the split (Cabantous *et al.* 2005). The two components must then be co-expressed to complement and yield fluorescence. wrmScarlet was recently redeveloped as a split fluorophore referred to as wrmScarlet _1-10_ (large fragment) and wrmScarlet _11_ (tiny polypeptide tag) (Goudeau *et al.* 2021). We cloned wrmScarlet _11_ into the SEC system for versatile N- or C-terminal tagging of any protein of interest, and this plasmid is now available via Addgene as pGLOW63 ( Fig. 1A ). As with other SEC plasmids (Dickinson *et al.* 2015), an in-frame epitope tag is included (in this case 3xMyc) to allow purification or immunostaining of the tiny-tagged target protein. We confirmed that the epitope tag did not disrupt the interaction of the split fluorophore by examining the endogenous expression of tiny-tagged HIS-72 in an *muIs252* ( *Peft-3::wrmScarlet _1-10_* ) genetic background (Goudeau *et al.* 2021). Indeed, the complemented RFP was visible in all somatic nuclei via epifluorescence ( Fig. 1B, top ).

Currently available *wrmScarlet _1-10_* genetic backgrounds were created with MosSCI + *unc-119 ( ed3 )* rescue and/or are limited to somatic-, germline-, or muscle-specific expression (Goudeau *et al.* 2021). Thus, we used CRISPR/Cas9 knock-in to create an alternative somatic *wrmScarlet _1-10_* construct (i.e. *P his-72 ::wrmScarlet _1-10_* ) that eliminates the need for *unc-119 ( ed3 )* rescue, as well as a construct expressed only in the nervous system ( *P rab-3 ::wrmScarlet _1-10_* ) ( Fig. 1B ). Finally, to allow highly versatile *wrmScarlet _1-10_* expression, we created a plasmid for easy cloning of any PCR-amplified promoter upstream of *wrmScarlet _1-10_* via Gibson assembly. This plasmid allows SEC-based CRISPR knock-in of *wrmScarlet _1-10_* at the safe harbor *ttTi5605* transposon site on LGII, and it is available via Addgene as pGLOW119 ( Fig. 1A ).

To investigate the potential of tiny tagging, we opted to tag three widely-studied proteins that are either small – EGL-1 (106 aa) and HIS-72 (151 aa) – and/or not fully functional when tagged with conventional full-length fluorophores ( EGL-1 and PTL-1 ) (Krieg *et al* . 2017). We find that these tiny-tagged proteins appear to be fully functional, but in most cases we were unable to observe the expression of the endogenous tiny tag *in vivo* via epifluorescence. We first investigated the pro-apoptotic protein EGL-1 . To confirm that tiny-tagged EGL-1 is functional, we examined the number of dopaminergic PDE neurons in L3 larval worms using a *Pdat-1::gfp* reporter (Davies *et al.* 2003). In *egl-1* null mutants, worms have additional PDE-like neurons as a result of blocked developmental cell death in the V5.paa neuronal lineage ( Fig. 1C ). Tiny-tagged EGL-1 animals have a wild-type number of PDE neurons, consistent with tiny-tagged EGL-1 being a fully functional protein ( Fig. 1D,E ). To visualize tiny-tagged EGL-1 expression, we exposed young adult worms to UV irradiation and screened for expression of the tag in pachytene germ cells undergoing DNA damage-induced apoptosis in the so-called “death zone” (Stergiou *et al.* 2007; Gartner *et al.* 2008). Unfortunately, we were unable to observe tiny-tagged EGL-1 expression anywhere in the germline of UV-treated worms at the timepoint *egl-1* mRNA levels are known to peak (6-12 hours post-UV, Stergiou *et al* . 2007) ( Fig. 1F ). Although use of transgenic *egl-1* reporter genes has been successful (e.g. Johnsen and Horvitz 2016), we suspect that endogenous *egl-1* expression is too transient or limited to observe *in vivo* . We next investigated PTL-1 , the ortholog of the human tau/MAP2 protein implicated in Alzheimer’s disease. *ptl-1* has a somewhat complex gene structure encoding four isoforms of PTL-1 , so we opted for an N-terminal tiny tag that tags three of the four isoforms ( Fig. 1G ). We call this set of proteins wrmScarlet _11_ :: PTL-1 (N). To test the functionality of tiny-tagged PTL-1 (N), we examined sensitivity to gentle touch because loss of proper PTL-1 function has previously been reported to impair gentle touch sensation (Gordon *et al* . 2008). The six neurons which mediate this sensation are particularly enriched with *ptl-1* , making them attractive targets to investigate. As with EGL-1 , tiny-tagging did not appear to disrupt the function of PTL-1 ( Fig. 1H ), but we were unable to observe tiny-tagged PTL-1 (N) expression in any mechanosensory neurons ( Fig. 1I ). This result was unexpected, but perhaps N-terminal tiny tags do not complement as well a C-terminal tiny tags or the fraction of PTL-1(N) complemented with wrmScarlet _1-10_ is not enriched enough at microtubules to observe. Overall, our findings suggest that tiny tags are generally innocuous enough to not disrupt the function of low MW endogenous proteins, but that the split fluorophore system is often not sensitive enough to allow observable expression of the complemented proteins via endogenous tags.

Overall, an important goal of the Glow Worms undergraduate research stream is to create and share resources with the *C. elegans* research community. Here, we have created several new plasmids and worm strains useful for CRISPR/Cas9 fluorescent protein knock-in. We anticipate that by expanding the CRISPR reagent toolkit, Glow Worms can have a meaningful and lasting impact on basic biological research, as well as foster a commitment to open and collaborative science in our students at a very early stage of their academic careers.

## Methods

***Plasmid construction***

pGLOW39 was constructed via Gibson assembly of PCR fragments amplified from parent vectors pDD287 (SEC^ccdB^AmpR^ccdB backbone; Addgene #70685) and pMS050 (mScarlet-I-C1; Addgene #91826). pGLOW63 was constructed via Gibson assembly of PCR fragments from parent vector pGLOW39 and the *wrmScarlet _11_* tiny tag sequence was cloned into the plasmid via PCR primer. pGLOW119 was constructed via Gibson assembly of PCR fragments amplified from parent vector pAP087 ( *ttTi5605* ^SEC^ccdB^mKate2 backbone) and worm gDNA from strain CF4582 ( *wrmScarlet _1-10_* ). All primer sequences available by request.

***Cloning homology arms into pGLOW39 or pGLOW63***

pGLOW39 (N-terminal)

**Table:** 

Digest (removes ccdB)	NgoMIV / ClaI
Gibson assembly sequence 5’ fwd primer	gtcacgacgttgtaaaacgacggccagtcg
Gibson assembly sequence 5’ rev primer	TTGATGACGGCCTCTCCCTTGGAGACCAT
Gibson assembly sequence 3’ fwd primer	GAGCAGAAGTTGATCAGCGAGGAAGACTTG
Gibson assembly sequence 3’ rev primer	tcacacaggaaacagctatgaccatgttat

pGLOW39 (C-terminal)

**Table:** 

Digest (removes ccdB)	NgoMIV / AvrII
Gibson assembly sequence 5’ fwd primer	gtcacgacgttgtaaaacgacggccagtcg
Gibson assembly sequence 5’ rev primer	CATCGATGCTCCTGAGGCTCCCGATGCTCC
Gibson assembly sequence 3’ fwd primer	GAGCAGAAGTTGATCAGCGAGGAAGACTTG
Gibson assembly sequence 3’ rev primer	ggaaacagctatgaccatgttatcgatttc

pGLOW63 (N-terminal)

**Table:** 

Digest (removes ccdB)	NgoMIV / ClaI
Gibson assembly sequence 5’ fwd primer	gtcacgacgttgtaaaacgacggccagtcg
Gibson assembly sequence 5’ rev primer	TTCTCGTATTGCTCGACGACGGTGTACAT
Gibson assembly sequence 3’ fwd primer	GAGCAGAAGTTGATCAGCGAGGAAGACTTG
Gibson assembly sequence 3’ rev primer	tcacacaggaaacagctatgaccatgttat

pGLOW63 (C-terminal)

**Table:** 

Digest (removes ccdB)	NgoMIV / AvrII
Gibson assembly sequence 5’ fwd primer	gtcacgacgttgtaaaacgacggccagtcg
Gibson assembly sequence 5’ rev primer	CATCGATGCTCCTGAGGCTCCCGATGCTCC
Gibson assembly sequence 3’ fwd primer	GAGCAGAAGTTGATCAGCGAGGAAGACTTG
Gibson assembly sequence 3’ rev primer	ggaaacagctatgaccatgttatcgatttc

***Cloning a promoter into pGLOW119***

**Table:** 

Digest (removes ccdB)	SpeI
Gibson assembly sequence fwd primer	ATGATGGTAGCAAACTCACTTCGTccggca
Gibson assembly sequence rev primer	CTTGATGACTGCTTCTCCCTTCGATACCAT

***Cloning the his-72 and rab-3 promoters into pGLOW119***

For *his-72* , a 1000 bp promoter was amplified from N2 gDNA with fwd primer 5’ ATGATGGTAGCAAACTCACTTCGTccggcaaaacgttatagtgtggacacca 3’ and rev primer 5’ CTTGATGACTGCTTCTCCCTTCGATACCATtgttgttctggaaattgagaattga 3’. For *rab-3* , a 1776 bp promoter was amplified from N2 gDNA with fwd primer 5’ ATGATGGTAGCAAACTCACTTCGTccggcacttgtcagtgtgaaccatgc 3’ and rev primer 5’ CTTGATGACTGCTTCTCCCTTCGATACCATctgaaaatagggctactgtaga 3’.

***Making strains GLW59 and GLW70***

SEC-based CRISPR/Cas9 (Dickinson *et al* . 2015) was used to insert all constructs into the genome. DNA was prepared and injected as previously described (Huang *et al* . 2021). The *his-72 ::wrmScarlet _11_* construct was injected into a *P his-72 ::wrmScarlet _1-10_* genetic background (strain GLW55) to make GLW59 [ *his-72 ::wrmScarlet _11_ ; P his-72 ::wrmScarlet _1-10_* ]. GLW59 was crossed into a *P rab-3 :: wrmScarlet _1-10_ ::SEC (* Rol) genetic background (strain GLW66) followed by removal of the SEC via heat shock excision to make GLW70 [ *his-72 ::wrmScarlet _11_ ; P rab-3 :: wrmScarlet _1-10_* ]. The *wrmScarlet _1-10_* constructs were targeted for CRISPR/Cas9 insertion at *ttTi5605* on LGII via plasmid pDD122 (Addgene #47550). Insertions were verified by PCR genotyping and complemented *his-72 ::wrmScarlet _11_* expression. Cas9/sgRNA and FP^SEC repair plasmids (see Reagents) available by request.

***Making strains GLW29 and GLW49***

The initial EGL-1 tiny tag strain was made by injecting the *egl-1 ::wrmScarlet _11_* construct into an *muIs252 Peft-3:: wrmScarlet _1-10_* genetic background (strain CF4582 ) to make GLW29 . This EGL-1 tiny tag was then crossed into other genetic backgrounds such as *egIs1 [Pdat-1::GFP]* (strain BZ555 ) to make GLW31 or *glh-1 (sam140[ glh-1 ::T2A::wrmScarlet _1-10_ ])* (strain DUP237) to make GLW71. The initial PTL-1 (N) tiny tag strain was made by injecting the *wrmScarlet _11_ :: ptl-1 (N)* construct into an *muIs252 Peft-3::wrmScarlet _1-10_* genetic background (strain CF4582 ) to make GLW49. This PTL-1 (N) tiny tag was then crossed into the *P rab-3 :: wrmScarlet _1-10_* genetic background (strain GLW96) to make GLW97. The *egl-1* and *ptl-1(N)* tiny tag edits were verified by PCR genotyping and Sanger sequencing. Cas9/sgRNA and FP^SEC repair plasmids (see Reagents) available by request.

***Epifluorescence imaging of Pdat-1::gfp, as well as his-72 and ptl-1(N) split fluorophores***

Epifluorescence microscopy was done on an Olympus BX51-F upright microscope using a 40x oil objective, Thorlabs CS2100M camera, and Micromanager/Fiji software. **BZ555** *egIs1 [Pdat-1::GFP]* , **JPS165** *egIs1 [Pdat-1::GFP]; egl-1 ( ok1418 )* , and **GLW31** *egIs1 [Pdat-1::GFP];* *muIs252 [Peft-3::wrmScarlet _1-10_ ]; egl-1 (utx23 [ egl-1 ::wrmScarlet _11_ ])* worms were collected as L3 larvae and mounted for microscopy using 10 mM levamisole. *Pdat-1::gfp* + PDE neurons were easily observed and counted. Split fluorophore strains were collected as adults and mounted for microscopy using a 3% agarose pad and 10 mM levamisole. We were not able to observe fluorescence of the PTL-1(N) tiny tag using the system available in our laboratory.

***Confocal imaging of the egl-1 split fluorophore following UV irradiation***

Synchronized young adult worms of strain GLW71 *glh-1 (sam140[ glh-1 ::T2A::wrmScarlet _1-10_ ]); egl-1 (utx23 [ egl-1 ::wrmScarlet _11_ ])* were irradiated with 100 J/m ^2^ UV-C using a Stratalinker 1800 as previously described (Stergiou *et al* . 2007). The presence of dead embryos on the plate at 24 hours post-UV was used as a positive control that UV treatment was yielding DNA damage. At 6-12 hours post-UV, adult worms were mounted for iSim confocal microscopy using a 60x oil objective and Kinetix camera. We were unable to observe split wrmScarlet complementation and fluorescence using this system.

***Analysis of mechanosensation in ptl-1 genetic backgrounds***

Assays for gentle touch sensitivity were done as previously described (Chalfie *et al* . 2014). Briefly, each worm was touched (i.e. tapped) a total of 5 times on the head and 5 times on the tail to trigger posterior or anterior movement, respectively. A total of 20 worms were assayed for each genotype. If the worm moved following a tap, the worm was sensitive to that tap. Data represents the average number of times (out of 5) the worm responded to a tap.

## Reagents

Plasmids

**Table:** 

Plasmid	Construct / Function	Location
pGLOW30	*Pmyo-2::mScarlet* / RFP array marker	Addgene
pGLOW31	*Pmyo-3::mScarlet* / RFP array marker	Addgene
pGLOW39	*mScarlet-I-C1* CRISPR knock-in / FP^SEC repair	Addgene
pGLOW63	* wrmScarlet _11_* CRISPR knock-in / FP^SEC repair	Addgene
pGLOW65	*egl-1* Cas9/sgRNA	Glow Worms
pGLOW66	*egl-1* FP^SEC *wrmScarlet _11_* C-terminal tag	Glow Worms
pGLOW73	*ptl-1 (N)* Cas9/sgRNA	Glow Worms
pGLOW77	*Pmyo-2::mNeonGreen* / GFP array marker	Addgene
pGLOW79	*Pmyo-3::mNeonGreen* / GFP array marker	Addgene
pGLOW87	*his-72* Cas9/sgRNA	Glow Worms
pGLOW88	*his-72* FP^SEC *wrmScarlet _11_* C-terminal tag	Glow Worms
pGLOW90	*ttTi5605 ^P his-72 ^wrmScarlet _1-10_ ^ tbb-2* (3'UTR)	Glow Worms
pGLOW105	*ptl-1 (N)* FP^SEC *wrmScarlet _11_* N-terminal tag	Glow Worms
pGLOW119	*ttTi5605 ^ccdB^wrmScarlet _1-10_ ^SEC^ttb-2* (3'UTR)	Addgene
pGLOW120	*ttTi5605 ^P rab-3 ^wrmScarlet _1-10_ ^ tbb-2* (3'UTR)	Glow Worms

Strains

**Table:** 

Strain	Genotype	Location
GLW27	* muIs252 [Peft-3::wrmScarlet _1-10_ :: unc-54 3'UTR + Cbr-unc-119 (+)] II; unc-119 ( ed3 ), his-72 (utx21 [ his-72 ::wrmScarlet _11_ ::3xMyc]) III *	CGC
GLW29	* muIs252 [Peft-3::wrmScarlet _1-10_ :: unc-54 3'UTR + Cbr-unc-119 (+)] II; unc-119 ( ed3 ) III; egl-1 (utx23 [ egl-1 ::wrmScarlet _11_ ::3xMyc]) V *	CGC
GLW31	* muIs252 [Peft-3::wrmScarlet _1-10_ :: unc-54 3'UTR + Cbr-unc-119 (+)] II; unc-119 ( ed3 ) III; egl-1 (utx23 [ egl-1 ::wrmScarlet _11_ ::3xMyc]) V; egIs1 [Pdat-1::GFP] *	Glow Worms
GLW49	* muIs252 [Peft-3::wrmScarlet _1-10_ :: unc-54 3'UTR + Cbr-unc-119 (+)] II; ptl-1 (utx41 [wrmScarlet _11_ ::3xMyc:: ptl-1 (N)]), unc-119 ( ed3 ) III *	Glow Worms
GLW55	* utxIs2 [P his-72 ::wrmScarlet _1-10_ :: tbb-2 (3'UTR)] II *	CGC
GLW59	* utxIs2 [P his-72 ::wrmScarlet _1-10_ :: tbb-2 (3'UTR)] II; his-72 (utx21 [ his-72 ::wrmScarlet _11_ ::3xMyc]) III *	Glow Worms
GLW66	* utxIs3 [P rab-3 ::wrmScarlet _1-10_ ::SEC:: tbb-2 (3'UTR)] II *	Glow Worms
GLW70	* utxIs4 [P rab-3 ::wrmScarlet _1-10_ :: tbb-2 (3'UTR)] II; his-72 (utx21 [ his-72 ::wrmScarlet _11_ ::3xMyc]) III *	Glow Worms
GLW71	*glh-1 (sam140[ glh-1 ::T2A::wrmScarlet _1-10_ ]) I; egl-1 (utx23 [ egl-1 ::wrmScarlet _11_ ::3xMyc]) V *	Glow Worms
GLW96	* utxIs4 [P rab-3 ::wrmScarlet _1-10_ :: tbb-2 (3'UTR)] II *	CGC
GLW97	* utxIs4 [P rab-3 ::wrmScarlet _1-10_ :: tbb-2 (3'UTR)] II; ptl-1 (utx41 [wrmScarlet _11_ ::3xMyc:: ptl-1 (N)]) III *	Glow Worms
